# Identification of Three Monofunctional Diterpene Synthases with Specific Enzyme Activities Expressed during Heartwood Formation in Western Redcedar (*Thuja plicata*) Trees

**DOI:** 10.3390/plants9081018

**Published:** 2020-08-12

**Authors:** Sifat Tasnim, Regine Gries, Jim Mattsson

**Affiliations:** Department of Biological Sciences, Simon Fraser University, 8888 University Drive, Burnaby, BC V5A 1S6, Canada; sifat_tasnim@sfu.ca (S.T.); margret_gries@sfu.ca (R.G.)

**Keywords:** diterpene synthase, heartwood, secondary metabolites, western redcedar

## Abstract

Upon harvest, Western redcedar (WRC; *Thuja plicata*) trees have a high incidence and extent of heartwood rot. While monoterpenoids and lignans have been linked to rot resistance in this species, other specialized metabolites, such as diterpenes, are likely to contribute to rot resistance. Here we report the cloning and functional assessment of three putative diterpene synthase (TpdiTPS) genes expressed during heartwood formation in WRC. The predicted proteins of the three genes lack either of the two catalytically independent active sites typical of most diTPS, indicating monofunctional rather than bifunctional activity. To identify potential catalytic activities of these proteins, we expressed them in genetically engineered *Escherichia coli* strains that produce four potential substrates, geranylgeranyl diphosphate (GGDP), *ent, syn,* and normal stereoisomers of copalyl diphosphate (CDP). We found that TpdiTPS3 used GGDP to produce CDP. TpdiTPS2 used normal CDP to produce levopimaradiene. TpdiTPS1 showed stereoselectivity as it used normal CDP to produce sandaracopimaradiene and *syn*-CDP to produce *syn*-stemod-13(17)-ene. These genes and protein enzymatic activities have not been previously reported in WRC and provide an opportunity to assess their potential roles in heartwood rot resistance in this economically important species.

## 1. Introduction

Western redcedar (WRC, giant arborvitae, *Thuja plicata* Donn ex D.Don) is a long-lived tree in the Cupressaceae family of conifers. It is common in the coastal and interior rainforests of Pacific Northwest America [1]. WRC lumber is among the most highly priced softwoods. In British Columbia, Canada, the WRC industry generates more than $1 billion annually, despite providing less than 10% of the total cut volume [2]. WRC wood is durable, swells marginally upon wetting, and is easy to work. Coastal natives have known these traits for millennia, using WRC for housing, canoes, carving of totem poles, and more [3]. The same traits make it a popular choice for roof and wall covering, decking, and garden furniture [4]. Paradoxically, considering the durability of WRC wood products, it has the highest incidence of heartwood rot of all Canadian softwood species, and up to 30% of harvested log volumes are culled due to extensive rot [5]. Since specialized metabolites, also known as secondary metabolites, are known to play a key role in heartwood rot resistance, identification of such metabolites and related genes could be used in selection for rot-resistance in WRC. 

Specialized metabolites are produced in the remaining living cells in the sapwood as the developmentally regulated sapwood to heartwood transition occurs [6,7,8,9]. These living cells are primarily ray cells, but also specialized epithelial cells lining resin ducts in Pinaceae species and axial xylary parenchyma cells in Cupressaceae species [8,10,11,12,13]. Specialized metabolite production can also be triggered prematurely closer to the bark in response to wounding and pathogen attack, sealing off healthy sapwood from the affected site [14,15]. Physiological studies of the transition zone show a temporary spike in respiration, loss of starch reserves, production of ethylene gas, enzymatic activities linked to the biosynthesis of specialized metabolites followed by the loss of nuclear integrity and cell death [16,17,18]. 

Specialized metabolites extracted from WRC have been studied for some time. Polyphenolic lignans are abundant in the WRC heartwood and include plicatic acid, plicatin, thujaplicatin and thujaplicatin methyl ether, and seven other compounds at low quantities [19,20,21]. Lignans have fungistatic activity [22]. Plicatic acid is a radical scavenger and metal chelator [23], most likely via the catechol group, also found in other lignans [24]. While the concentration of plicatic acid and an unknown lignan correlate with rot resistance of wood in service [19], similar correlations have not been found to date for rot resistance in growing trees. The second main group of extractives in WRC heartwood is ring-expanded monoterpenoid-tropolones, known as thujaplicin (in α, β, γ isoforms), β-thujaplicinol, β-dolabrin, thujic acid, methyl thujate and trace amounts of nezukone, and thujin [19,20,21]. Heartwood extractive analysis in second growth WRC trees collected from coastal and interior region of British Columbia has shown significantly higher levels of tropolone (e.g., β-thujaplicin) content in coastal population, compared to the interior population where more decay in heartwood has been reported [25]. Tropolones degrade over time and appear not to play a role in wood in service [19]. In addition to tropolones, the monoterpenes carvacrol and carvacrol methyl ether have been identified in WRC heartwood extracts [26]. However, there are reasons to believe that other terpenes are present in WRC heartwood. For example, the heartwood of the related species *Thuja occidentalis* L. contain several sesquiterpene alcohols [27], and heartwood of more distantly related species, such as *Juniperus chinensis* L., harbor many different diterpenes [28]. 

Diterpenes are biosynthesized by the cyclization of geranylgeranyl diphosphate by diterpene synthases and the subsequent oxidation by cytochrome P450-dependent monooxygenases. Diterpenes constitute the second largest class of terpenes, with over 2200 compounds belonging to 130 distinct skeletal types [29]. Diterpenes have been shown to confer resistance against pests and pathogens in several species, including rice (*Oryza sativa* L.), maize (*Zea mays* L.) [30], Pinaceae species [31], tobacco [32], and *Arabidopsis thaliana* (L.) Heynh [33]. Depending on domain structure, active sites, and signature motifs, there are three major classes of diTPSs: monofunctional class I and class II diTPSs and bifunctional class I/II diTPSs [34,35]. Class II diTPSs contain conserved DXDD motif critical for protonation-initiated cyclization of geranylgeranyl diphosphate (GGDP) into the bicyclic prenyl diphosphates *ent*-copalyl diphosphate (*ent*-CDP), normal-CDP, or *syn*-CDP [36,37]. In class I diTPSs, conserved DDXXD motif in the C-terminal domain is required for the diphosphate ionization-initiated cyclization and rearrangement of CDP to diterpene hydrocarbons [38]. Bifunctional class I/II diTPSs combine both reactions in a single protein where CDP intermediate freely diffuse from class II to the class I active site [39]. The function of diTPS enzymes can be altered by the steric and electrostatic control of the intermediate carbocations formed through substrate protonation (class II) or ionization (class I) [40,41]. A single amino acid substitution can lead to the redirection of diTPS product specificity, highlighting the functional plasticity of diTPSs and their capacity to evolve new functions [42,43,44,45]. Diterpenes are typically found as carboxylic acids in conifers, better known as diterpene resin acids [31]. Biochemical studies provide evidence for modification of diterpenes first by hydroxylation into alcohols followed by oxidation into aldehydes and acids [46]. Several conifer cytochrome P450-encoding genes have been cloned and shown to carry out diterpene alcohol oxidations into diterpene carboxylic acids [29,46,47,48,49,50]. The structural diversity of diterpene resin acids results from the combined activities of multienzyme families of diterpene synthases and cytochrome P450 monooxygenases that produce various tricyclic carboxylic acids, such as abietic, dehydroabietic, isopimaric, levopimaric, neoabietic, palustric, pimaric, and sandaracopimaric acids. Others produce bicyclic diterpene acids, such as agathic, isocupressic, and *trans*-communic acids [31,34,51]. Different methods of extractions are required to separate different types of polar and non-polar terpenoids. For example, the extraction of more polar resin acids, such as abietic, dehydroabietic, neo-abietic, palustric, pimaric, and isopimaric acid, is not entirely possible from hexane or other non-polar solvents and requires derivatization before Gas Chromatography (GC) analysis [12,52,53,54]. In *Pinus radiata* D. Don, the dominant resin components include diterpene resin acids, fats, fatty acids, sterols, and phenols where resin acids are concentrated in the inner heartwood, and fatty acid esters remain at a high proportion in the sapwood [55,56,57].

To date, no gene has been identified in WRC with a potential role in the biosynthesis of diterpenes during heartwood formation in WRC. Here we described three cDNAs encoding three putative diterpene synthases that are highly upregulated in the sapwood (SW) to heartwood (HW) transition zone. We also characterized their enzymatic activities, revealing hitherto unknown functions in this species.

## 2. Results

### 2.1. Identification of Three Putative Diterpene Synthases Expressed in WRC Sap to Heartwood Transition Zone

**Figure 1:**
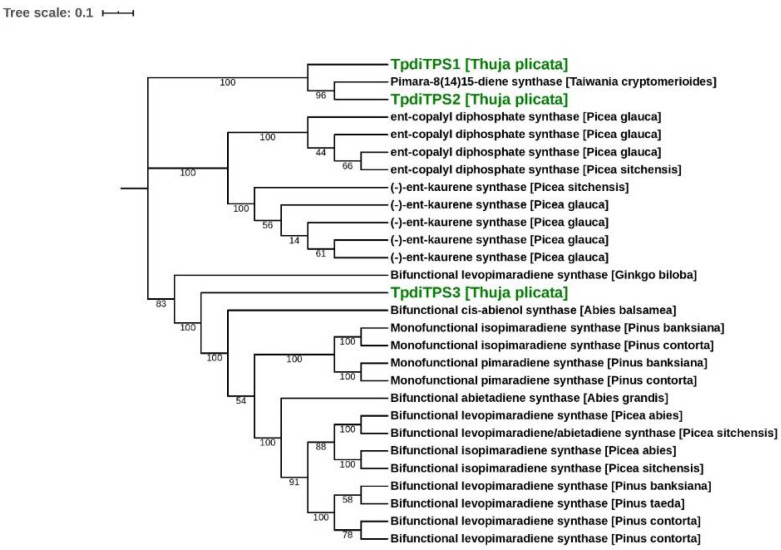
Phylogenetic tree of conifer diterpene synthases. Phylogenetic analysis was performed with 28 full-length diterpene synthases. Proteins identified in this study are highlighted in green. TPS (Terpene Synthases) of the Cupressaceae, except for the TPdiTPS3 cluster apart from TPS of the Pinaceae, indicative of the independent diversification and evolution of specific TPS functions in the Cupressaceae and the Pinaceae. Scale bar indicates 0.1 amino acid substitution per site. The number shows bootstrap confidence values from 100 replicates.

To identify putative diterpene synthases, we searched a transcriptome derived from WRC tree stem tissues with a putative diterpene synthase annotation. We identified three contiguous complementary DNA (cDNA) sequences (Supplementary Material Figure S1) and named them temporarily *TpdiTPS1–3*. The contiguous cDNA sequences of *TpdiTPS1, 2,* and *3* were 2731 bp, 2898 bp, 3080 bp, along with predicted open reading frames encoding 846, 879, and 865 amino acid residue proteins, respectively. The contigs had extensive read coverage at 5′ and 3′ ends (data not shown). The transcript sequences were confirmed by polymerase chain reaction (PCR) amplification from cDNA, cloning, and Sanger sequencing. A comparison of predicted TpdiTPS1, 2, and 3 protein sequences with that of known protein sequences showed a maximum of 74%, 80%, and 87% identity, respectively, across the length of the proteins to known conifer diterpene synthases (alignments in Supplementary Material Figures S2 and S3). A phylogenetic analysis, including conifer mono-, sesqui-, and diterpene synthases, also showed that TpdiTPS1–3 grouped with diterpene synthases (Supplementary Material Figure S4). Among conifer diterpene synthases, TpdiTPS1 and 2 separated at the base from diterpene synthases in the Pinaceae family of conifers to form a clade that grouped together with Pimara-8(14)15-diene synthase from *Taiwania cryptomerioides* Hayata (Figure 1). This grouping reflects current phylogenetic classifications as both WRC (*T. plicata*) and *T. cryptomerioides* belong to the Cupressaceae family of conifers. TpdiTPS3 fell in a separate clade together with diterpene synthases from Pinaceae species. Its position at the base of the clade indicates an early divergence from the Pinaceae diterpene synthases.

**Figure 2:**
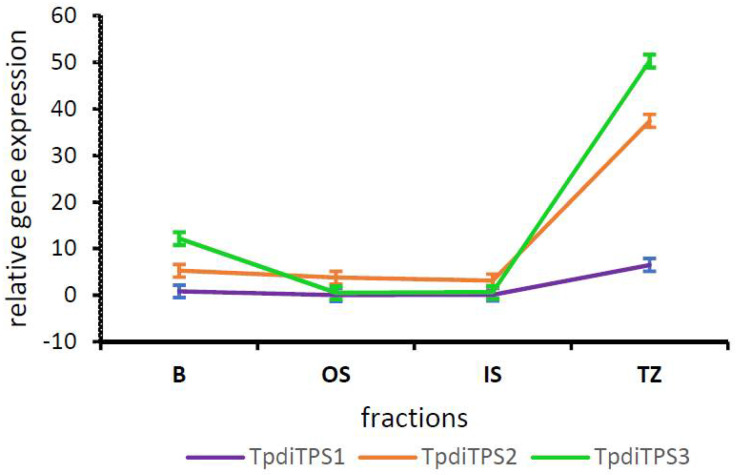
*TpdiTPS2* and *3* are expressed at elevated levels in the sap to heartwood transition zone. Expression of *TpdiTPS1, 2,* and *3* in RNA-seq libraries derived from four different fractions of Western redcedar (WRC) wood. B—bark, OS—outer sapwood, IS—inner sapwood, TZ—sapwood to heartwood transition zone. Error bars were calculated based on two biological replicates. TMM (trimmed mean of M-values) normalized TPM (transcripts per million) values were used to calculate relative expression levels. Statistical differences of expression in different fractions were determined in R (version 4.0.1) by two-way ANOVA and Tukey HSD tests.

Assessment of the expression in RNA-seq libraries derived from radial fractions of stems revealed high steady-state levels of transcripts in the heartwood transition zone relative to sapwood alone (Figure 2). The average expression of *TpdiTPS2* was 10.8 times higher and *TpdiTPS3* 86.7 times higher in the sap to heartwood transition zone relative to their expression in sapwood only (*p* < 0.05). Although the mean *TpdiTPS1* expression was higher in the transition zone relative to the sapwood fractions, it was not statistically significant. *TpdiTPS3* transcript levels were also elevated in the bark fraction relative to the sapwood fractions (*p* < 0.05).

**Figure 3:**
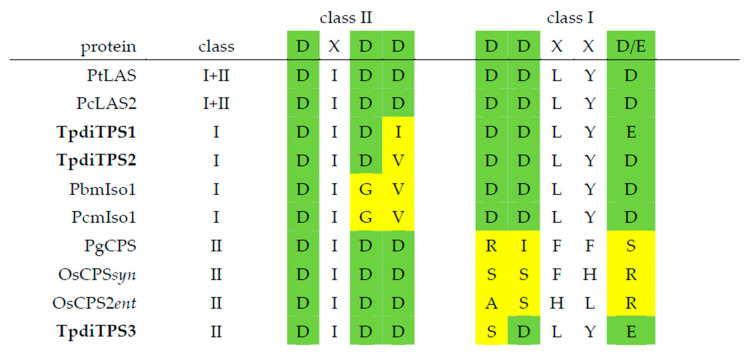
Putative catalytic motifs in TpdiTPS1 and 2 match monofunctional class I proteins and TpdiTPS3 motifs match monofunctional class II proteins. Amino acid alignments of class I and II motifs of *Thuja plicata* TpdiTPS (1–3) with *Pinus taeda* bifunctional class I/II levopimaradiene synthase (PtLAS, Q50EK2), *Pinus contorta* bifunctional class I/II levopimaradiene/abietadiene synthase2 (PcLAS2, JQ240311), *Pinus banksiana* (PbmIso1, JQ240313), and *Pinus contorta* (PcmIso1, JQ240314) monofunctional class I isopimaradiene synthase1, *Picea glauca* monofunctional class II *ent*-copalyl diphosphate synthase (PgCPS, GU045755), *Oryza sativa* monofunctional class II *syn*-copalyl diphosphate synthase (OsCPS*syn*, AY530101), *Oryza sativa* monofunctional class II *ent*-copalyl diphosphate synthase (OsCPS2*ent*, AY602991). Amino acids that differ from the conserved class II DXDD and class I DDXXD/E are shaded in yellow. Full-length alignments are shown in Supplementary Material Figures S2 and S3.

To identify possible functions, we compared the putative catalytic motifs in TpdiTPS1, 2, and 3 (Figure 3) with the DXDD motif in the N-terminal domain indicative of a class II terpene synthase and a DDXXD motif in the C-terminal domain indicative of a class I terpene synthase [58,59]. TpdiTPS1 and 2 contained the class I motif DDXXD/E in the C-terminal domain but lacked the class II DXDD motif in the N-terminal domain. Instead, they contained DXDI and DXDV, respectively. These results suggest that TpdiTPS1 and 2 are monofunctional class I enzymes that cannot use GGDP as a substrate but can use CDP as a substrate for diterpene cyclisation. The lack of type II activity was confirmed by *in vitro* assays, which showed that recombinant TpdiTPS1 and 2 proteins could not use GGDP as a substrate to synthesize diterpenes (data not shown). In contrast to TpdiTPS1 and 2, TpdiTPS3 matched the class II DXDD consensus motif, but not the class I DDXXD/E motif. This pattern of motifs is typical of monofunctional type II proteins that can use GGDP to synthesize CDP but not carry out the final cyclisation to generate diterpenes.

### 2.2. Assessment of Potential Mono-Functional Class I Diterpene Synthase Activity in TpdiTPS1 and 2

**Figure 4:**
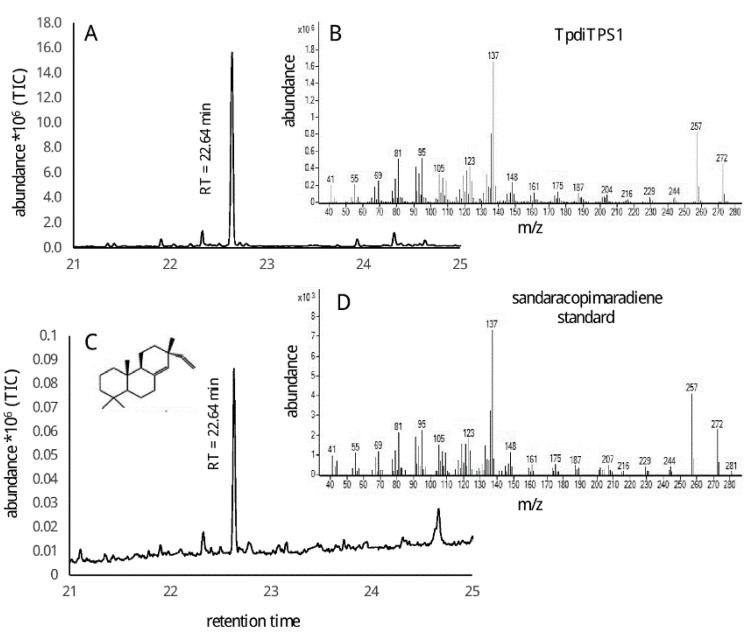
GC-MS analysis of sandaracopimaradiene produced by TpdiTPS1 synthase from normal- copalyl diphosphate (CDP); (**A**) = total ion chromatogram (TIC), (**B)** = mass spectrum of sandaracopimaradiene, (**C**,**D**) = TIC and mass spectrum of an authentic standard of sandaracopimaradiene produced by *Aspergillus niger* sandaracopimaradiene synthase (AnCPS-PS) [61].

**Figure 5:**
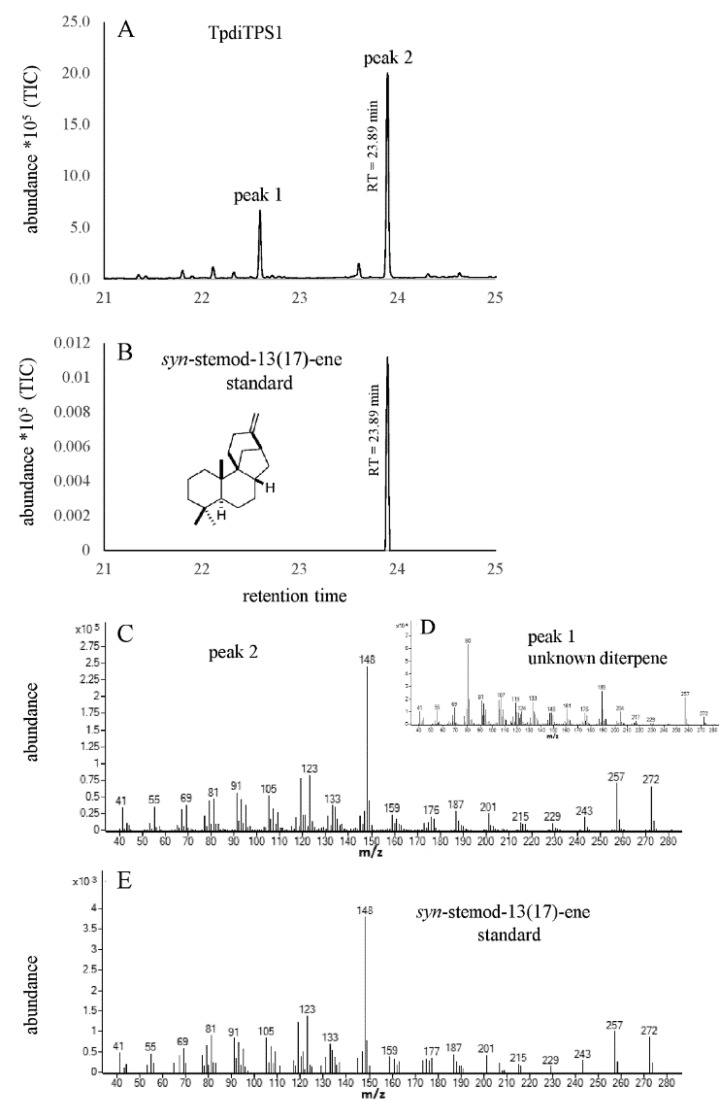
(**A**) = GC-MS total ion chromatogram (TIC) showing unknown diterpene (peak 1) and *syn*-stemod-13(17)-ene (peak 2) produced by TpdiTPS1 from *syn*-CDP. (**B**) = TIC of *syn*-stemod-13(17)-ene standard produced by *Oryza sativa syn*-stemod-13(17)-ene synthase (OsKSL11) [60]. (**C**–**E**) = mass spectra of peak 2, peak 1, and an authentic *syn*- stemod-13(17)-ene standard produced by OsKSL11 [60], respectively.

The lack of an intact DXDD motif in the class II active site of TpdiTPS1 and TpdiTPS2 suggests that these proteins may require CDP rather than GGDP as a substrate. The majority of labdane-related diterpenoid natural products are derived from the *ent*, *syn*, or normal stereoisomers of CDP. As CDP is not commercially available and expensive to chemically synthesize, we used a previously described *in vivo* co-expression system in *E. coli* [60] in which plasmid-based expression of the known proteins result in strains that produce GGDP, *ent*-CDP, *syn*-CDP, and normal-CDP. In these strains, we co-expressed TpdiTPS1, 2, or 3 to test the possibility of bifunctional or monofunctional enzyme activities. The major diterpene product from the co-expression of TpdiTPS1 in a strain that produces normal-CDP was sandaracopimaradiene (Figure 4). When expressed in a strain that generates *syn*-CDP as a substrate, TpdiTPS1 produced *syn*-stemod-13(17)-ene as the major product with an unknown diterpene as a minor product (Figure 5). Expression of TpdiTPS1 in strains that produce GGDP or *ent*-CDP did not yield any products (data not shown).

**Figure 6:**
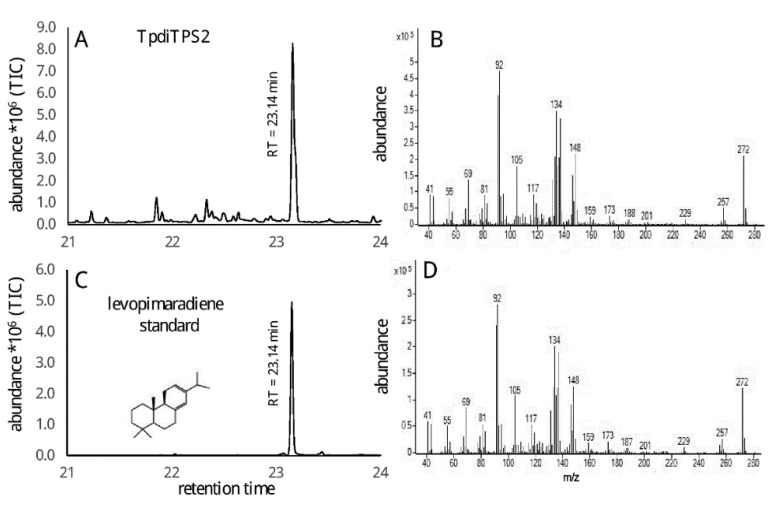
GC-MS analysis of levopimaradiene produced by TpdiTPS2 from normal-CDP. (**A**) = total ion chromatogram (TIC), (**B**) = mass spectrum of levopimaradiene, (**C**,**D**) = TIC and mass spectrum of an authentic standard of levopimaradiene produced by *Abies grandis* abietadiene synthase (AgAS) [63].

Expression of TpdiTPS2 with normal-CDP produced levopimaradiene as a major product but did not catalyze any product when incubated with GGDP, *ent*-CDP, and *syn*-CDP (Figure 6). The mass spectrum was identical to the levopimaradiene produced by *Picea abies* levopimaradiene/abietadiene synthase (PaTPS-LAS) [62]. 

### 2.3. Monofunctional Class II TpdiTPS3 Synthesize the Intermediate CDP

**Figure 7:**
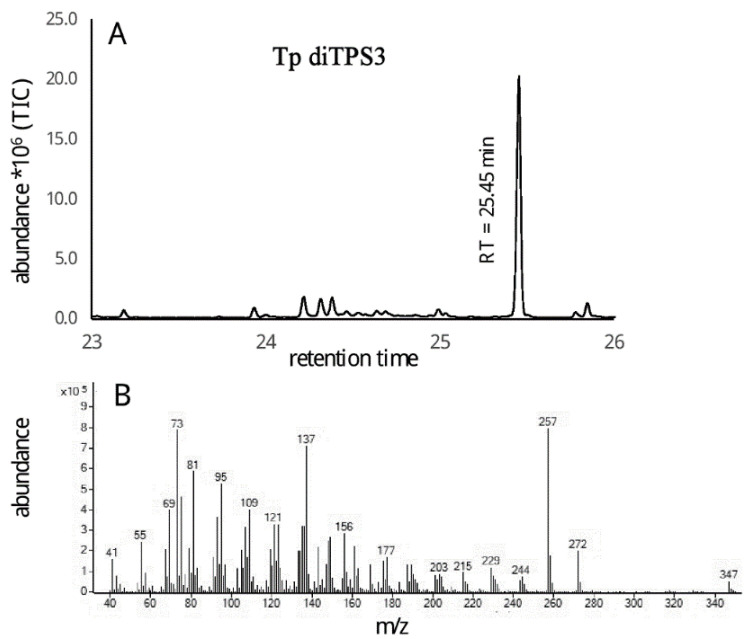
(**A**) = GC-MS analysis of copalol derived from geranylgeranyl diphosphate (GGDP) by TpdiTPS3, (**B**) = Mass spectrum of the chromatograph peak for TpdiTPS3.

**Figure 8:**
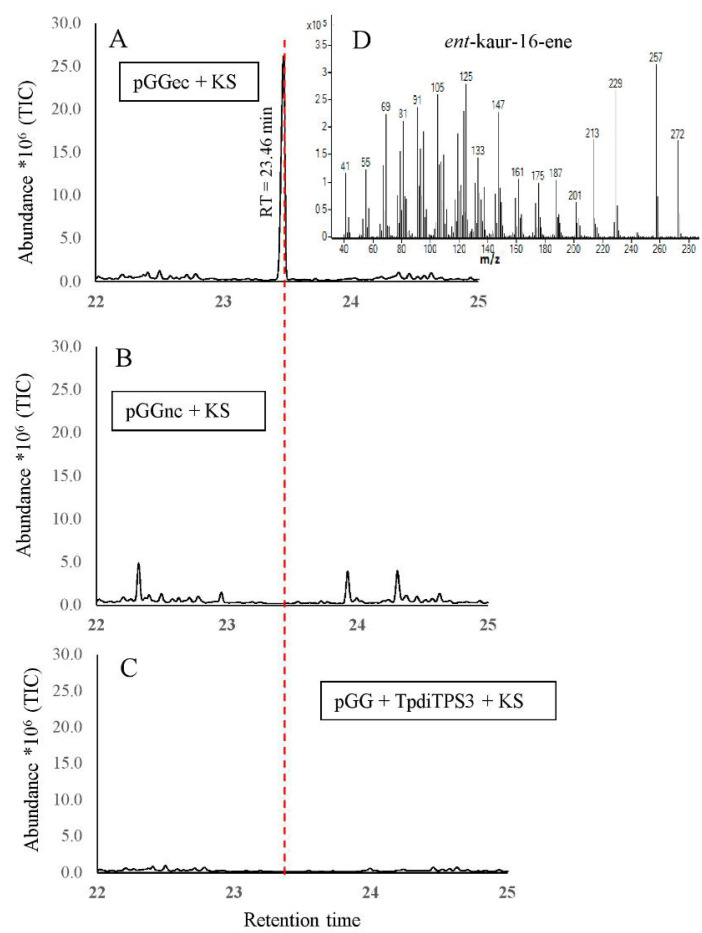
Confirmation of enantiospecific enzymatic product of TpdiTPS3. (**A**) = Expression of *ent*-CDP synthase with *ent*-specific kaurene synthase results in *ent*-kaur-16-ene biosynthesis. (**B**) = Expression of *ent*-specific kaurene synthase with normal CDP synthase did not yield any *ent*-kaur-16-ene. (**C**) = Expression of *ent*-specific kaurene synthase with GGDP synthase and TpdiTPS3 also did not yield *ent*-kaur-16-ene. (**D**) = Mass spectrum of *ent*-kaur-16-ene.

TpdiTPS3 resulted in copalol when co-expressed with the gene producing GGDP (Figure 7), in agreement with being a monofunctional class II enzyme. No product was generated when expressed together with genes that produce *ent*-CDP, *syn*-CDP, and normal-CDP. The TpdiTPS3 product and *syn*-CDP had different retention times and mass spectra. Since *ent* and normal CDP are enantiomers, they are not separated by GC-MS analysis. Therefore, to assess whether the product of TpdiTPS3 is *ent* or normal CDP, we used the *A. thaliana* AtKS (*ent*-kaur-16-ene synthase) enzyme [60] that only reacts with *ent*-CDP to produce *ent*-kaur-16-ene as a means of testing substrate specificity. While expression of an *ent*-CDP synthase with the *ent*-kaur-16-ene synthase resulted in the production of the expected *ent*-kaur-16-ene (Figure 8A), the combination of TpdiTPS3 and *ent*-kaur-16-ene synthase did not (Figure 8C), providing evidence by exclusion that TpdiTPS3 is a normal-CDP synthase.

## 3. Discussion

**Figure 9:**
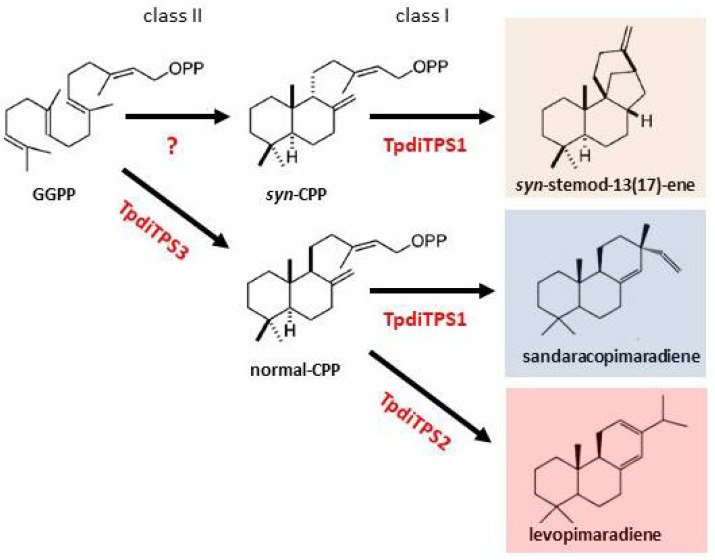
Model of the monofunctional diterpene biosynthesis pathway potentially active during heartwood formation in WRC trees based on the results in this study. The question mark indicates that an unidentified *syn*-CDP synthase may also be active to provide an alternative substrate for TpdiTPS1.

The genetic basis of terpene biosynthesis during heartwood formation in Western redcedar (WRC) is currently unknown. Here we show that three cDNAs derived from mRNAs expressed in the sap to heartwood transition zone of WRC trees, named TpdiTPS1, 2, and 3, encode diterpene synthases with specific in vivo activities. Consistent with the catalytic motif profiles in their peptide sequences, they proved to be mono rather than bifunctional diterpene synthases. TpdiTPS3 had a DXDD motif in the N-terminal domain known to be required for the protonation-initiated cyclization of GGDP to CDP [37] but deviated from the DDXXD motif in the C-terminal domain, known to be required for the diphosphate ionization-initiated cyclization and rearrangement of CDP to diterpene hydrocarbons [64]. Accordingly, the expression of TpdiTPS3 in the presence of GGDP resulted in the biosynthesis of CDP. A method of exclusion was used to gain insight into what isomer of CDP is produced. The retention time and mass spectrum matched that of normal and *ent*-CDP (indistinguishable from each other) but differed from and excluded *syn*-CDP. Since an *ent*-CDP-specific *ent*-kaurene synthase did not produce *ent*-kaurene when expressed with TpdiTPS3 but did when expressed with an *ent*-CDP synthase, it appears that TpdiTPS3 produces normal-CDP; see model, Figure 9. Confirmation by an independent method, i.e., chiral column chromatography, is currently beyond our means. However, normal-CDP identity is also consistent with the normal-CDP use of TpdiTPS1 and 2. A class II normal-CDP synthase has been identified before in maize, wheat, red sage, and the conifer *T. cryptomerioides* [65,66,67,68], which, like WRC, belongs to the Cupressaceae family of conifers. There are also known bifunctional diterpene synthases that produce normal-CDP as an intermediate [39,62,63,69,70,71].

The peptide sequences of TpdiTPS1 and 2 lacked an intact N-terminal DXDD motif but contained the C-terminal DDXXD motif required for the rearrangement of CDP to diterpene hydrocarbons, typical of class I diterpene synthases [38,64]. In agreement with their motif profile, both TpdiTPS1 and 2 produced diterpenes when co-expressed with proteins providing CDP. TpdiTPS1 accepted normal and *syn*-CDP as a substrate, but not *ent*-CDP. TpdiTPS1 produced sandaracopimaradiene in the presence of normal-CDP and *syn*-stemod-13(17)-ene in the presence of *syn*-CDP. The use of *syn*-CDP suggests that a *syn*-CDP synthase may be expressed during WRC heartwood formation. On the other hand, if *syn*-CDP is not available, TpdiTPS1 may be limited to normal-CDP and produce only sandaracopimaradiene during heartwood formation. Normal-sandaracopimaradiene has been found as the single product from one monofunctional class I diterpene synthases cloned from rice [72]. In conifers, monofunctional class I diterpene synthases have been found to produce normal-sandaracopimaradiene only as minor products (*Pinus banksiana* Lamb.; 10%, *P. contorta*; 12%) [73], which differs from the >90% normal-sandaracopimaradiene produced by TpdiTPS1. Bifunctional diterpene synthases with normal-sandaracopimaradiene as minor product have been found in *Picea sitchensis* (Bong.) Carrière and as a major product in the fungi *Aspergillus niger* Tiegh. [61,63,74]. A stemodene synthase has been identified in plants only once before [75], in rice. TpdiTPS2 used normal-CDP to produce normal-levopimaradiene. An enzyme with the same specificity was recently identified in another Cupressaceae species, *T. cryptomerioides* [64]. Bifunctional diterpene synthase that produces levopimaradiene as one of several products have been found in *Ginkgo biloba* L. and the conifers *Abies grandis* Douglas ex D. Don, *Pinus taeda* L. and *Picea abies* (L.) Karst. [62,63,70,71]. However, there is evidence that the four diterpenes identified in *in vitro* assays of the *P. abies* enzyme are epimers from a thermally unstable diterpenol [62,76]. It is possible that we obtained similar artifacts, although we did not see a similar spread of artifact products. Taken together, the substrate and product specificities of TpdiTPS1, 2, and 3 are unusual among plant species and provide the first model framework for diterpene biosynthesis during heartwood formation in WRC (Figure 9). 

To date, no single diterpene or diterpenoid has been described in WRC tissues [19,20,23,24,26,77,78,79,80,81], so it is unclear in what modified form we should expect to find the three diterpene products identified in this study. In the closely related species Thuja standishii (Gordon) Carrière, extracts from stem bark contain both diterpene alcohols and acids [82], none with the same hydrocarbon skeleton as described in this study. Among other Cupressaceae, the sandarac tree *Tetraclinis articulata* (Vahl) Mast. is well known for its gum, used since antiquity as a natural pictorial and wood varnish [83]. Sandaracopimaric acid and sandaracopimarinol are the two most abundant diterpenoids in sandarac gum [84]. Species in the Callitris genus have also been used for the production of sandarac gum and also have alcohol and acid forms of sanadaracopimaradiene in bark resin [85,86]. 

Little is known about the toxicity of the diterpenes identified here and their potential natural modified forms. Sandaracopimaric acid, purified from pine cones, inhibits both brown and white rot-causing fungal species in petri-dish experiments. Of nine tested diterpene acids, levopimaric acid generated the strongest growth inhibition of brown and white rot fungal species [87]. Closely related compounds are also found among diterpene phytoalexins in Poaceae species, produced in response to microbial attack [88,89]. Tests demonstrate their toxicity to a range of fungal and bacterial species [90,91,92]. There is also evidence that related diterpene acids play a role in conifer resistance to insect pests and associated fungi [93]. 

## 4. Materials and Methods 

### 4.1. Materials

RNA was extracted from approximately 60-year-old trees taken down at the Simon Fraser University campus. Cut sections were frozen in liquid nitrogen and radial fractions were taken using chisel and hammer. The following fractions were taken; bark to cambium excluding most dead outer bark, ~2 cm sapwood, ~2 cm older sapwood, and transition zone consisting of two annual rings of sapwood and two rings of heartwood. Sticks of samples were freeze-dried for 3 days, milled to ≤0.5 mm particle size. To recover low concentration of RNA, approximately 1.5 g powder was rehydrated in ice-cold extraction buffer (2% CTAB; 200 mM Tris, pH 8.0; 50 mM EDTA, pH 8.0; 1 M NaCl; 0.5% activated charcoal; 1.5% polyvinylpolypyrrolidone; and 1.5% β-mercaptoethanol) ground in liquid nitrogen and transferred to Oakridge centrifuge tubes. After evaporation of nitrogen, 10 mL pre-warmed (65 °C) extraction buffer was added, mixed, and tubes were incubated in a water bath (65 °C) for 10 min. Proteins and cell wall material was extracted twice with an equal volume of chloroform (13,000 Relative Centrifugal Force (RCF) at the bottom). The aqueous phase was mixed with an equal volume of 100% isopropanol and kept at −20 °C for 2–3 h to precipitate nucleic acids. After centrifugation at 17,000 RCF at the bottom of the tubes, the pellet was resolubilized in 0.5 mL SSTE (1 M NaCl; 0.5% SDS; 10 mM Tris-HCl, pH 8.0; and 1 mM EDTA, pH 8.0) buffer followed by chloroform extraction and RNA precipitation by addition of ¼ volume of 10 M LiCl overnight on ice at 4 °C in 1.5 mL tubes. After 30 min centrifugation at 14,000 RCF, the pellet was washed in 75% ice-cold ethanol, dried, and resuspended in nuclease-free water. RNA integrity was analyzed by bleach gel electrophoresis [94]. Residual DNA was removed, and 1st strand cDNA synthesized (AccuRT genomic DNA removal, EasyScript^TM^ cDNA synthesis kit, abmgood.com).

### 4.2. Selection of Candidate Genes

Three contiguous cDNA sequences encoding putative *Thuja plicata* diterpene synthases (*TpdiTPS1-3*) were selected based on expression in an RNA-seq library made from the transition zone where heartwood was formed (Tasnim, Wiggins, Mattsson in manuscript). The transcript sequences were confirmed by polymerase chain reaction (PCR) amplification from cDNA, cloning, and Sanger sequencing. The accession numbers were for TpdiTPS1-3, MT468207, MT468208, and MT468209, respectively. BLASTX and BLASTP (National Center for Biotechnology Information) were used to assess sequence similarity to other known diTPS sequences and the presence of conserved RRX_8_W, DXDD and DDXXD motifs. The protein sequences were aligned by the Clustal Omega program and formatted in Gene Doc. Chloroplast transit peptide in the Open Reading Frames (ORFs) of the selected TPS genes was identified with iPSORT and TargetP1.1., and deleted during plasmid cloning.

### 4.3. Cloning of diTPS cDNAs

Two sets of primers were designed to amplify the full-length cDNA (Supplementary Material Table S1), keeping in mind that the vector would be linearized with *Ssp*I restriction enzyme (New England Biolabs, Ipswich, USA). The online Expasy translate tool was used to double-check the continuity of the ORF-His tag fusion protein. A nested polymerase chain reaction (PCR) was performed to amplify three predicted full length TPS cDNAs with the first set of primers according to CloneAmp^TM^ HiFi PCR premix (Takara Bio, Kusatsu, Japan) protocol with a 55 °C annealing temperature. PCR products were diluted 10-fold in water and used for PCR using a second set of primers and 60 °C annealing temperature. Resulting single-band products were cloned into *Ssp*I-digested pETHis6GST expression vector (Addgene) by the Sequence and Ligation Independent Cloning method (SLIC; [95]). The mix was transformed into Stellar chemically competent cells (Takara Bio, Kusatsu, Japan). Colonies with recombined plasmid were identified by colony PCR, used for plasmid purification. Inserted fragments were fully sequenced by Sanger, confirming expected nucleotide sequences. All resulting recombinant proteins contained the open reading frame with an N-terminal His tag.

### 4.4. Transformation and In Vivo Coexpression

Recombinant protein expression, purification, and assays were performed as described [60]. For the expression of putative terpene synthase genes, plasmid carrying the gene of interest together with individual pGG, pGGeC, pGGsC, pGGnC plasmids was sequentially transformed into BL21 Star^TM^ (DE3, ThermoFisher, Waltham, USA) chemically competent *E. coli* cells. To confirm the stereochemistry of normal-CPS activity, TpdiTPS3 was co-expressed with pGG and a class I labdane related diterpene synthase, i.e., *Arabidopsis thaliana* kaurene synthase (AtKS) that only reacts with *ent*-CPP. AtKS, cloned in pDEST15/rAtKS [60], was also co-expressed individually with pGGec and pGGnc to confirm the presence and absence of *ent*-kaur-16-ene. To express TPS proteins, four independent colonies of transformants were grown in 5 mL Luria-Bertani medium supplemented with 35 µg/mL kanamycin and 25 µg/mL chloramphenicol and cultured overnight at 37 °C, at 225 rpm. The following day, the bacterial cell suspensions were diluted 1:50 with Terrific Broth medium containing the same antibiotics and grown as above until an OD_600 nm_ of 0.6. Cultures were then shifted to 16 °C for 1 h before induction with 0.5 mM IPTG (isopropyl-1-thio-beta-D-galactopyranoside) addition of 5 g/L glycerol, and growth for ~72 h at 16 °C, at 220 rpm. 

### 4.5. Isolation of Recombinant Protein Products

The bacterial cells were collected by centrifugation at 1400 *g* for 30 min. The hydrocarbon product was extracted from the media with an equal volume of hexane with 2% (*v/v*) ethanol [96,97]. 

### 4.6. Silica and Alumina Chromatography

A Pasteur pipette was loaded with glass wool plug, ~0.6 g silica gel, ~0.1 g MgSO_4,_ and washed five times with hexane. The organic phase (8 mL) was passed over the silica gel column. In the case of oxygen-containing products, the media was extracted with an equal volume of hexane: ethyl acetate (6:1), and the organic phase was passed through a Pasteur pipette with glass wool plug and ~0.6 g basic alumina column overlaid with ~0.1 g (NH_4_)_2_SO_4_. Approximately 8 mL of the eluate was evaporated under an N_2_ stream. The sample was resuspended in 500 µL of hexane and derivatized overnight at room temperature with *N*,*O*-bis(trimethylsilyl)trifluoroacetamide (MilliporeSigma, Burlington, USA) before GC-MS analyses. 

### 4.7. GC-MS Analysis

GC-MS analysis was performed on an Agilent 7890B gas chromatograph and a 5977A MS Detector at 70 eV (Agilent Technologies, Santa Clara, USA). One microliter of the extract was injected, and compounds were separated on a DB5 column (30 m × 0.25 mm ID, 0.25 μm film) with an He flow of 1 mL/min. The inlet was operated at 250 °C, splitless mode. The GC temperature program was as follows: 50 °C, hold 5 min, and 10 °C/min ramp to 280 °C, 10 min hold. Diterpenes were identified by comparing retention time and mass spectra with standards kindly provided by Drs. Reuben J. Peters and Meimei Xu, Iowa State University, USA. The retention indices of the identified compounds are provided in Supplementary Material Table S2.

## 5. Conclusions

The study aimed to identify genes encoding diterpene synthases that are likely to contribute to heartwood rot resistance in WRC. From our results, we can conclude that there are at least three putative diterpene synthases expressed in the region in which specialized metabolites accumulate to generate heartwood. The enzymes are monofunctional and together provide a potential pathway for the production of levopimaradiene, sandaracopimaradiene, and *syn*-stemod-13(17)-ene from GGDP via the intermediates normal and *syn*-CPP. These are the first terpene synthases identified as active during heartwood formation in WRC and provide a starting point to (1) identify corresponding diterpenoids in heartwood extracts, (2) identify genes that carry out required modifications to the hydrocarbon skeletons, and (3) carry out Single Nucleotide Polymorphism-heartwood rot association studies to test their potential roles in heartwood rot resistance, the single most important economic problem in this species [1,3,5].

## Supplementary Materials

The following figures and tables are available online at https://www.mdpi.com/2223-7747/9/8/1018/s1. Figure S1: Nucleotide and predicted amino acid sequences of three *Thuja plicata* diterpene synthases described in this study. Figure S2: Amino acid alignment of *Thuja plicata* monofunctional diTPS1 (TpdiTPS1) and diTPS2 (TpdiTPS2) with *Pinus taeda* bifunctional levopimaradiene synthase (PtLAS, Q50EK2), *Pinus contorta* bifunctional levopimaradiene/abietadiene synthase (PcLAS2, JQ240311), *Pinus banksiana* (PbmIso1, JQ240313), and *Pinus contorta* (PcmIso1, JQ240314) monofunctional class I isopimaradiene synthase1. Figure S3: Amino acid alignment of *Thuja plicata* monofunctional diTPS3 (TpdiTPS3) with *Picea glauca* monofunctional *ent*-copalyl diphosphate synthase (PgCPS, GU045755), *Oryza sativa syn*-copalyl diphosphate synthase (OsCPS_syn_, AY530101) and *Oryza sativa ent*-copalyl diphosphate synthase (OsCPS2_ent_, AY602991). Figure S4: Phylogenetic relationships of conifer terpene synthases. The number of bootstrap replications was 100. The abbreviation of species are as follows: *Physcomitrella patens* (Pp) was used to root the tree. *Picea glauca* (Pg), *Picea sitchensis* (Ps), *Picea abies* (Pa), *Physcomitrella patens* (Pp), *Abies grandies* (Ag), *Pinus taeda* (Pt), *Pinus contorta* (Pc), *Pinus banksiana* (Pb), *Taiwania cryptomerioides* (Tc), *Abies balsamea* (Ab), *Chamaecyparis obtuse* (Co), *Chamaecyparis formosensis* (Cf), *Pseudotsuga menziesii* (Pm), *Cycas taitangensis* (Ct). Table S1: Primers used for nested PCR and SLIC-based cloning into expression plasmid. Table S2: Kovats Retention indices of identified diterpene compounds.
