# Supplementary material for: Identification of Three Monofunctional Diterpene Synthases with Specific Enzyme Activities Expressed during Heartwood Formation in Western Redcedar (Thuja plicata) Trees
Source: Plants (Basel). 2020 Aug 12;9(8):1018. doi: 10.3390/plants9081018 (PMC7464036; doi:10.3390/plants9081018)
Supplement: Supplementary file 1 [file plants-09-01018-s001.pdf]

Supplemental figures

Identification of three monofunctional diterpene synthases with specific enzyme activities expressed during heartwood formation in Western redcedar (*Thuja plicata*) trees

Sifat Tasnim, Regine Gries and Jim Mattsson

>TpdiTPS1 (MT468207)

```
CTTCTAATGCCTACTGAAACAAATTTGATCTCTATTTTATCAAAATGGCTCAGAAGATGTTTTCTCCGTCCATTTCA
GTTTCCAAGTCTCGAGGATGGATTTCAACCAAAACCTCTGGATTTTCACCCATTGGAAAGTACTCGCGCTCAAAGAG
TCTGGCTTGCCACAACATGCCATCTGCTGTTGTTGTTGGAGAGGATGCTAAGACTCTTCAGGCTTTGAGAATAGCGC
ATAAGGAGAGTAAAATCAATCCTAATCACGCGAAACCAGATTATGTTCACTCTGTTTCAACATTTGAGGAGGCACCC
TTTGATGAGATGGATAAGAGAATAGAGGAATTGGTTACAGAGATCAAAGGGCTTTTTAATTCGATGGAAGATGGGGA
AATAAGTCCCTCTGCTTACGATACTGCGTGGGTAGCGAGAGTTCATGCCATTGATGGCTCTGTTAAACCCCAATTTC
CTCAAATGGTGGACTGGATTCTTCAAAATCAGTTACCGGACGGTTCTTGGGGAGAAAAGAGTCGCTTTTTAGCGTGT
GATAGACTGCTCAATACTCTTTCCTGCTTAGTTACTCTCTCCATCTGGGGCGTTGGAAACAATCAAGTGAACAGGGG
TCTTAATTTCTTGAGGAGAAATACAGAAGGAATGATTAAAGAAGCACTCGGTCATCATCAACCCAAGGGATTCGACA
TGGTCTTTCCTGTACTGCTGAATGAAGCCAAACTTTTGGGTTTGGATCTTCCTTATGGGCTATATATCGTTAAACAA
ATACGCGAAAAGCCAGACTTGGAATTGAAAAAAGTATTTGTTGAAGAGCTACACGGTCATCCCTCAACAATGTTACA
GTGTCTGGAAGGCGTACAAGAAATAATTGATTGGAAAAGGGTCCTGAAACTGCAATCCAAGGATGGATCTTTCTCTG
GCTCGCCAGCATCTACAGCTTGCGTATTTATGCACACAGGAGACATTAAATCTCTCCAATTTTTGACGAGCCTTGTT
AAAAAGTTTGGAGACCATGTGCCGAGCATGTATCCGGTGGATATAGCGGAGCGTTTAAGAGCCGTGGATTGCGTTGA
GCGTCTTGGACTCGAACGCCATTTTCAAACGGAAATTAAACAAGCAATGGATTATGTGTTCCAGTACTGGAGTGAAA
GAGGCATTGGATTTGGAAGGGAAAGCCTGGTTCCTGATATTGATATTACAGCCACTGCCTTCAGGCTTCTCAGAACC
TTCGGCTACTCTGTATCTTCCGAGGTTCTGCAAAACATTAAAGCCGAGGCTGAAGAACTTCTTAAGCTGTCTGGTAA
CGAAAACAGCGCAGGAATAATCGGAATATTGAGCCTGTATAGATCTTCACAGCTTAACTTTCCGGGCGAAATTGTAA
TGAAGGAAATAGATTGCTTTGCAAAAGATTACCTGGCTGAATTCCTGCAAACCAAGAACTTTTCTCAGGTGAAGGTT
GTCAAGGAAAACCTTCCCGAAGAGGTTGAATATGCTTTGTCTGCTCAATGGAATAGAAATATGCCAAGGCTGATGTC
TAGAAATCAGATAGAATTGTTTAATCCCAATGACCTATGGCTGGGGAAGACATTATATCACCTGCCAAATGCTAGCA
ATGACAAGTATTTAGAATTGGCCAAACTCGACTTCAACCGCATTCAGGCTACACACAGATTTGAAATACAACGAATA
CAGAGATGGTACAAGGATTGCAATTTCCCGCGGTTGGATTTTACTCGTCACAGAGAAGTGGCAGTTTACTGGACCTC
TTCAGCGGTGATGTTTGAACCACAATACACCGATTGCAGACTCGATTACGCAAAAGCCGGACTCCTGGCTACTATCA
CAGACGACCTCTATGAATCCTATGCAACTCTCAACCAACTCAAGCTTTTCAATGAAGCTTTCGAAAGATGGGATCCA
TTAATGAGCGAGCAACTGCCAGAGGACATGAAAATAGTTTTCATGGGAATCTATAACACTTTGACCGATATTTCTGA
GCGAGCACTGAAGGTTCAAGGTCGTGATGTGCTTCCTTACCTGCGCCAACAGTGGTTAAATCTGTTGTTCAGTTTCA
CAAAGGAAAGAGAGTGGATGGAAAGAAGTTATTCGCCATCATTGGATGAATACTGGGCGAATGCAGAAGTGTCGATA
GCACTGGAAACAACTATCCTGTCGCCAATATTTTCTACGGGAAATTTTCTTCCTGATCGCATTCTGGAGAAGATTAA
CTTTCTGGATCTGGTCAGTCAAACAGGGCGTCTTATGAACGACGTTAGAACTTTCCAGAAGGAAAGAGATCGCGGGG
AATTGGCTTCATTCGTAGAATGCTACAAAAATGAGCTCCATGGATGCACTGAAGAAGAAGCCCTGAATTACATGGAG
AGAATGAACGAAGAGGCCCTTATCAATTTGAACTACCATTTTCTAATGCGATCAGACATACCTAAGTGCTACAGAAC
ACTTCTTTTCAACACGGCTAGGATAATGCAAATGATTTACAGGAAGGAAGACGGTTTTCGAAATGCCGCGGAGTACC
TCGAAGATTCAATTAAAAAATCTCTGTACGAACCAGTGCTGTAACAGATTACTATTCTGTAGTGTTGAAATTCGAAC
ATCAGTCAAATAAAAAAAATTATTCCAAATGTTAATAAAATATTTTTTATTACTAAAAAAGGCATCATTACAATCAT
GGTAATATTATTAGTTAATTTTCTGCAAAAAAAAAA
```

>TpdiTPS1

MAQKMFSPSISVSKSRGWISTKTSGFSPIGKYSRSKSLACHNMPSAVVVGEDAKTLQALRIAHKESKINPNHAKPDY
VHSVSTFEEAPFDEMDKRIEELVTEIKGLFNSMEDGEISPSAYDTAWVARVHAIDGSVKPQFPQMVDWILQNQLPDG
SWGEKSRFLACDRLLNTLSCLVTLSIWGVGNNQVNRGLNFLRRNTEGMIKEALGHHQPKGFDMVFPVLLNEAKLLGL
DLPYGLYIVKQIREKPDLELKKVFVEELHGHPSTMLQCLEGVQEIIDWKRVLKLQSKDGSFSGSPASTACVFMHTGD
IKSLQFLTSLVKKFGDHVPSMYPVDIAERLRAVDCVERLGLERHFQTEIKQAMDYVFQYWSERGIGFGRESLVPDID
ITATAFRLLRTFGYSVSSEVLQNIKAEAEELLKLSGNENSAGIIGILSLYRSSQLNFPGEIVMKEIDCFAKDYLAEF
LQTKNFSQVKVVKENLPEEVEYALSAQWNRNMPRLMSRNQIELFNPNDLWLGKTLYHLPNASNDKYLELAKLDFNRI
QATHRFEIQRIQRWYKDCNFPRLDFTRHREVAVYWTSSAVMFEPQYTDCRLDYAKAGLLATITDDLYESYATLNQLK
LFNEAFERWDPLMSEQLPEDMKIVFMGIYNTLTDISERALKVQGRDVLPYLRQQWLNLLFSFTKEREWMERSYSPSL
DEYWANAEVSIALETTILSPIFSTGNFLPDRILEKINFLDLVSQTGRLMNDVRTFQKERDRGELASFVECYKNELHG
CTEEEALNYMERMNEEALINLNYHFLMRSDIPKCYRTLLFNTARIMQMIYRKEDGFRNAAEYLEDSIKKSLYEPVL

>TpdiTPS2** (**MT468208)

GGAATCTAATTGAGATTTGGCAGGGTATGCCCTGTGCAGCTCCGATAAAACCCTCTCTCTCTCTACTCCCCATGGGA
TTCAAAGTCTTCTTCACAAGGCGACATCTAATTTGTTAAAAATGTCCCAGAGTTTGTGTCCGCGCCTCAGTTTGTTT
TCGAAGCCTACTACCAAATCCACACAACGTCTTTCCAATACTTCTCTGCCTTTTACAAACTACGCTCGCATTAAGAG
CATAGATTGCTACAACATGACATCTGCTCCTGCTCTTGGCGACAATGCGAAAACACTTCACGCTGCAGCGATTGCGC
ATCCGGAGCCCAAAATCTATCCTAATGCTGGGAAGCCAGATTATGTTCATTCTAATTCAACATTTGAGGAGGCACCG
TTGGAGGAGATGGATAAGCGAATAGAGGCATTGGTTGCGGAGATCAAAGAGCTGTTTTATTCAATGGAAGATGGGGA
AATAAGTCCTTCCGCATACGATACTGCGTGGGTAGCGAGAGTGCCTGCCATTGATGCCTCCGCTCAACCCCAATTTC
CCCAATTGCTGGACTGGATTCTTCAGAATCAGTTAGCGGACGGTTCCTGGGGCCAGCAGAGTCGCTTTTTAGCGTCT
GATAGGTTCCTCAATACTCTTGCCTGCCTCCTTACTCTCACCTTCTGGGGCGTTGGAAACAATCAAGTGCAGAGAGG
TCTTCATTTCTTAAGAGGAAATATGGAAGCAATGGTTAAAGAAGCTGTAGCATTCGGTCATCAAGGATTCGAGATGG
TTTTGCCTGCACTGTTGAATGAAGCCAAACATTTGGGCTTGGATCTTCCTTACGAGCTACCTATCATCCAGCAAATA
AACAAAAAGAGGGACTCCGAATTGAAAAAGGTATCTGTTGAGGAGCTACACACGCATCCGACAGCAATGTTGCAGTG
TCTGGAAAGCATACAAGAAGTAGTCGATTGGAAAGACATCCTGAAATTGCAATCGAAGGATGGGTCTTTCTCAGGCT
CGCCAGCATCTACAGCTTGTGTATTTATGCACACCGGAGATAAGAAATGCCTACGATTCTTGGCGGGTCTTGTTACA
AAGTTTGAAGACTATGTCCCCTGCATGTATCCAGTGGACATAGCAGAGCGTTTGAGGGCCGTGGATAGTGTTGAACG
TCTGGGGCTTGAACGCCATTTCCAAACGGAGATCAAACAAGCCTTGGACTATGTGTTCCAGTACTGGGGTGAAAGAG
GAGTTGGATTTGGAAGGGACAGCCTGGTTCCGGATATTGATGTCACAGCCACGGGCTTCAGGCTTCTCAGGATGTTC
GGCTACACTGTGTCTCCAGACTTTCTGCAAAACATCAAAGACGAAGCTGAAGAACTCTGTAAGCTGTCTGATGGTGA
AAACAGGGGAAGAGTAATCGACATGCTGAGCCTGTATAGATGCTCACAGATTAACTTTCCGGGAGAAAATGTAATGA
GAGAAATAGGTGCATTTGCCAAAGATTACTTGGCCGAATCCCTGCAAAGCAACAACTTTTCTCAGGCGACGGCTGTC
AAGGATAACCTTCGCCAAGAGGTCGAATATGCTTTGTTTGCCAGATGGAATAGAAATATGCCGAGACTGGTGATTAT
AAATAACATAGAAGTGTTTAATCCCGATGACTTATGGCTGGGGAAGACATTATATCAAATGCCAAATGCGAGCAACG
GCAAGTATTTAGAATTGGCCAAACTCGAGTTCAACCGCACTCAGGCTATACACAGATCCGAAATACAACATATTAAG
AGATGGTACAAGGCTTGCAATTTCCCCCAGCTGGAATTTACTCGTCACAGAGAAGTGGCAATCTACTGGACTGCGGC
AGCGGTAATGCCCGATCCCCAATACACCGACTGTAGACTCGCTTATGCAAAAGCAGGAATCATGGCTGTTATCACAG
ACGACTTGTATGACACCTGTGCAACTCTGGAGCAGGCCAAGCTCTTCAACGAAGCTTTTGAAAGGTCGCCGCGTTTA
ATCCCCATCTCAGTTAATGCGAATAAGGAATTGGAATCGGCGATATTAACATTGATTGTGTTTGATGGGTGCAGATG
GGATACAGAGCAAATCGAGCATCTACCAGAGGAGATGAGAATAGTATTTATGGGGCTGTACAACACTTTGAGGGAGA
TATCTGAAGGAGCGCGGGAGGTCCAAGGGCGTGATGTGCTTCCTTACCTGCGGCAAAAGTGGTTGGATCTGTTCAGG
AGATACACGAAAGAAACAGAGTGGATGGAAAGGAGGCATTCGCCGTCATTAGAAGAATACTGGGAGAACGCGGTGGA
GTCGATAGCACTGGGAGTCACTACCCTCACCCCAATATTCTCTACCCAAGATCTTCTTCCTGATCATCTCCTCCAGA
AATTTGACTTCCGCGCAGACTTTCTGAATCTCGTCAGTCTCACGGGGCGCCTCATTAACGACGTCAGAACTTTCCAG
GAGGAAAGAGATCGGGGGGAATTGGCTTCATGCGTGCAATGCTACAGAAATGATAATCCGGGATGCACGGAAGAAGA
AGCCCTGAATTATCTGTACGGGGTGAATGAGGACGCCCTGACTAAATTGAATTATCAGTTTTTGATGCGTGAAGACA
TTCCCAAGAGCTTCAGAACTGTTCTTTTCAACACGGCCAGGGTAATGCAATTGTTTTACAGGAACATTGACGGCTTT
CTAAATGCCGCCGAAGAGATGAAAGTCTTCATTAAAAAGACTCTCTATGAACCCCTGCTCTAATAAATTGCTCTATT
GGATGAACTGTGTAATAAATGAAATTCCATTTTTTTTTTAGTTTTTGGTGAATATATTTGAAGGAGAAACTTATTAA
TGTACACTTCAATTTATGAAAGGATAACGAATCAAAATTTACTATCATT

>TpdiTPS2

MSQSLCPRLSLFSKPTTKSTQRLSNTSLPFTNYARIKSIDCYNMTSAPALGDNAKTLHAAAIAHPEPKIYPNAGKPD
YVHSNSTFEEAPLEEMDKRIEALVAEIKELFYSMEDGEISPSAYDTAWVARVPAIDASAQPQFPQLLDWILQNQLAD
GSWGQQSRFLASDRFLNTLACLLTLTFWGVGNNQVQRGLHFLRGNMEAMVKEAVAFGHQGFEMVLPALLNEAKHLGL
DLPYELPIIQQINKKRDSELKKVSVEELHTHPTAMLQCLESIQEVVDWKDILKLQSKDGSFSGSPASTACVFMHTGD
KKCLRFLAGLVTKFEDYVPCMYPVDIAERLRAVDSVERLGLERHFQTEIKQALDYVFQYWGERGVGFGRDSLVPDID
VTATGFRLLRMFGYTVSPDFLQNIKDEAEELCKLSDGENRGRVIDMLSLYRCSQINFPGENVMREIGAFAKDYLAES
LQSNNFSQATAVKDNLRQEVEYALFARWNRNMPRLVIINNIEVFNPDDLWLGKTLYQMPNASNGKYLELAKLEFNRT
QAIHRSEIQHIKRWYKACNFPQLEFTRHREVAIYWTAAAVMPDPQYTDCRLAYAKAGIMAVITDDLYDTCATLEQAK
LFNEAFERSPRLIPISVNANKELESAILTLIVFDGCRWDTEQIEHLPEEMRIVFMGLYNTLREISEGAREVQGRDVL
PYLRQKWLDLFRRYTKETEWMERRHSPSLEEYWENAVESIALGVTTLTPIFSTQDLLPDHLLQKFDFRADFLNLVSL
TGRLINDVRTFQEERDRGELASCVQCYRNDNPGCTEEEALNYLYGVNEDALTKLNYQFLMREDIPKSFRTVLFNTAR
VMQLFYRNIDGFLNAAEEMKVFIKKTLYEPLL

>TpdiTPS3(MT468209)

CTCTTAACACCTATTCATTGAGGTCGCTGACAAATTTGAAAGGCAGGTGCAATTGGGACTTATCAGCTGAACAGTGC
AAAAGCTTAAAAACATACCAGTATTTTTTCTTCTTCTTCTTTTCTAACTTAGAGAAAATGGCTCAATCTTTGATTTC
TTCGGCCAATTCTTGTATTGCAAAGACTCGACTTTCAGATGTTAATGGCAGAACAGAGCTCAACAGATCTCGACCAT
TATCATTTTCATTTTCCTCCTCTAAGCTGGTGAAGAGGCAATGGAATAAGCAGTCAGCTGGTTTGGTTTTGGCTTGT
CTTGGAGATTCCAGGCGTTCTTCTTCCCCTGCTGCTACAGGGGCTGCCACTGCTTCTACATCGGTGAAACGAGAGTA
CCCTCCAGCAGTTTGGAATGATGACGTGATCAATTCTCTGATATCAACTTATAAATCTGCAGATGTAGCAGAGCAGG
AGAAAAGGTCTGAGACATTGATAGCGGAGATAAAGGGCATGTTTAAATCAATGGGGGATGGTGAAACGAATCCTTCT
GCCTATGATACTGCTTGGGTTGCAAGGATACCTGCTGTGGATGGCTCTAATGGCCCTCAGTTTCCTCAGACGCTTCA
ATGGATTCTGCAAAACCAGTTGAGCGATGGTTCCTGGGGCGAAGAATTGTGCTTCTTGACATATGACCGTGCTCTGG
CTACTCTTGCCTGTGTTATTACGCTCTCTCTGTGGAATACAGGGGAAGAACAAGTGAACAAAGGTGTTGAATTTATA
AAGAAACATGCTGAGCGAATGGAAGGAGAAGCGGACAATCACCGGCCAAGTGGATTCGAAATTGTTTTCATTTCAAT
GCTCAACGAAGCTAAGACTCTAGGATTGGATCTACCTTATGATCTGCCTTTCTTTAAGCAGATTAATGAAATGCGCG
AAACTAAGCTTAAAAAGATTCCTTTGAATGTTGTCCACGCCATTCATACAACAATTCTGTACTCGTTGGAGGGGTTG
CAGGAAATAATAGACTGGGACAAGATAATGAAGCTTCAATCCAAGGATGGATCCTTCCTTAGCTCCCCTGCTTCCAC
AGCAGCCGTGTTCATGCGCACAGGAGACAAAAAGTGCTTAGACTTTTTGAGCTTCGTTCTCAACAAATTTCAAGATC
ATGCTCCTTGTCACTATCCTCTTGATCTCTTCGAGCGTCTATGGGCTGTTGACACTTGCCAACGCCTGGGAATCGAT
CGCCATTTCAAAGACGAGATCAAAGATACTCTAGATTATGTCTACAGCTATTGGAACGATAGAGGCATCGGATGGGC
AAGAGAAAATGCAGTTGGTGATATTGATGATACTGTCATGGGCCTTCGCCTCCTCAGACTTAGCGGATACAATGTGT
CATCAGATGCACTGAAAACTTTCAGAGACGAGAATGGAGAATTCTTTTGCTTCATGGGTCAAACACAGAGAGGAGTT
ACAGACATGTTAAACGTTCATCGCGGCTCACAAGTTGCATATCCAGGCGAAACAGTCATGGAAGAAGCAAAACAGTG
CACGCTCAGATATTTGACCAGCGCTCTTGAAAATGTCGGCGCTTTTGACAAATGGGCCCTGAAAAAGGATCTTCAAG
GAGAGGTGGAATACGTGCTTAGATATCCATGGCATAGAAGCCTGCCAAGACTGGAGGCAAGGAACTACGTAGAACAG
TACGGTGCAAATGATGTGTGGCTTGGAAAGTCCATGTACCTGATGTATTACGTAAGCAATCAGAAATATTTAGAGCT
GGCCCAAATAGACTTCAACAAGGTTCAAGCGGTACACCAAAAGGAAATTCAGGAGCTTCAGAGGTGGTGGAAATCAT
CTGGTTTCACAAAGCTCAATTTCACGCCTGAACGTGTGGTTGAAATCTATTTTGGAGTGGCGGCGACCATGTTTGAG
CCAGAGCTAGCTACACTAAGAGCTGTTTACACCAAAACTTCAATCTTTTCTGTCATTCTCAGCGATCTCTATGAAGC
GCAGGGATCAACTCACAACATGGCGTTGTTCTCTGAAGCAGTCAAAAGATGGGATATGTCAATGGTTGACCGCATGC
CAGAAGAAATGAAAATCTGCTTCAAGGGCTTGTATGAAACAGTCAATGAATTCGCAGAGGAAGGAAGGAAGAGACAA
GGGCGTGACGTATTGCCTTACCTCCGAAACCTGTGGGAGGTTCAGTTGGAATCCTACACCAAGGAAGCAAAAATGGC
TCAGGCCAAATACATGCCTTCCTTCCAAGAGTACATGGAGAATGCCAAAGTATCAATGGGACTAGCCACCATAGTTT
TGACCTCAGCTCTCTTTACAGGAGATCTTCTATCTGATGAAACACTTTCCAAAATGGGCTATGATTCCAAATTTGTC
CAATTAATGGTTTCAACAGGGCGTTTGGTTTGTGATACAAAAACTTACCAGGAGAAACAAGAGAGAGGTCTCCCTTC
AGCTGTACAATGCTACATGAAAGACCATCCTGAAATCTCAGAAAAAGAAGCTTTAGACCATATCTACTCAGTACTGC
AGAATTCCCTGCTGGAATTGAATTGGGAACTTGTTAACAACAGAGAAATGCCAGAGACTTGTAGAAGCCTGGTTTTT
AACACAGCAAGAATAATGCAGCTATTTTACATGGAGAGAGATGGTTTTATTATGTCCCATCTAGAAATGCAACAACT

```
TGTGAGAAAGTGCCTTTTCGAGCCAGTGGCAGTATAGAAATCAAACTAGACAAAGACCAACATTTTTTTTTCTGTCT
TAGGCATACAATATCCATGATGGGTCTCTCAGAAACTACCTTTTTTCTCGCAAAATTCTTTCTTTTTTCTAGGATAC
TTCAGACTTCCATCAATTCGCTGCAAAAATAAATGCAAGTTGTATAGATTAGGTGAATCTACTCTTGCCTGATATGA
CCCTCACTTGGAGAGGGATTTGGGAAGACTTTACACTCACATATGTGGGTTTTATTTACGCTAGTTATGTAAATTTC
CATACTAGTTTGAATAATTGTTATACATATTCACATCAAAATATTTTGGTGAACATTTTTATATAAGTCCAAGCTCT
```

>TpdiTPS3

```
MAQSLISSANSCIAKTRLSDVNGRTELNRSRPLSFSFSSSKLVKRQWNKQSAGLVLACLGDSRRSSSPAATGAATAS
TSVKREYPPAVWNDDVINSLISTYKSADVAEQEKRSETLIAEIKGMFKSMGDGETNPSAYDTAWVARIPAVDGSNGP
QFPQTLQWILQNQLSDGSWGEELCFLTYDRALATLACVITLSLWNTGEEQVNKGVEFIKKHAERMEGEADNHRPSGF
EIVFISMLNEAKTLGLDLPYDLPFFKQINEMRETKLKKIPLNVVHAIHTTILYSLEGLQEIIDWDKIMKLQSKDGSF
LSSPASTAAVFMRTGDKKCLDFLSFVLNKFQDHAPCHYPLDLFERLWAVDTCQRLGIDRHFKDEIKDTLDYVYSYWN
DRGIGWARENAVGDIDDTVMGLRLLRLSGYNVSSDALKTFRDENGEFFCFMGQTQRGVTDMLNVHRGSQVAYPGETV
MEEAKQCTLRYLTSALENVGAFDKWALKKDLQGEVEYVLRYPWHRSLPRLEARNYVEQYGANDVWLGKSMYLMYYVS
NQKYLELAQIDFNKVQAVHQKEIQELQRWWKSSGFTKLNFTPERVVEIYFGVAATMFEPELATLRAVYTKTSIFSVI
LSDLYEAQGSTHNMALFSEAVKRWDMSMVDRMPEEMKICFKGLYETVNEFAEEGRKRQGRDVLPYLRNLWEVQLESY
TKEAKMAQAKYMPSFQEYMENAKVSMGLATIVLTSALFTGDLLSDETLSKMGYDSKFVQLMVSTGRLVCDTKTYQEK
QERGLPSAVQCYMKDHPEISEKEALDHIYSVLQNSLLELNWELVNNREMPETCRSLVFNTARIMQLFYMERDGFIMS
HLEMQQLVRKCLFEPVAV
```

Supplemental figure S1. Nucleotide and predicted amino acid sequences of three *Thuja plicata* diterpene synthases described in this study.

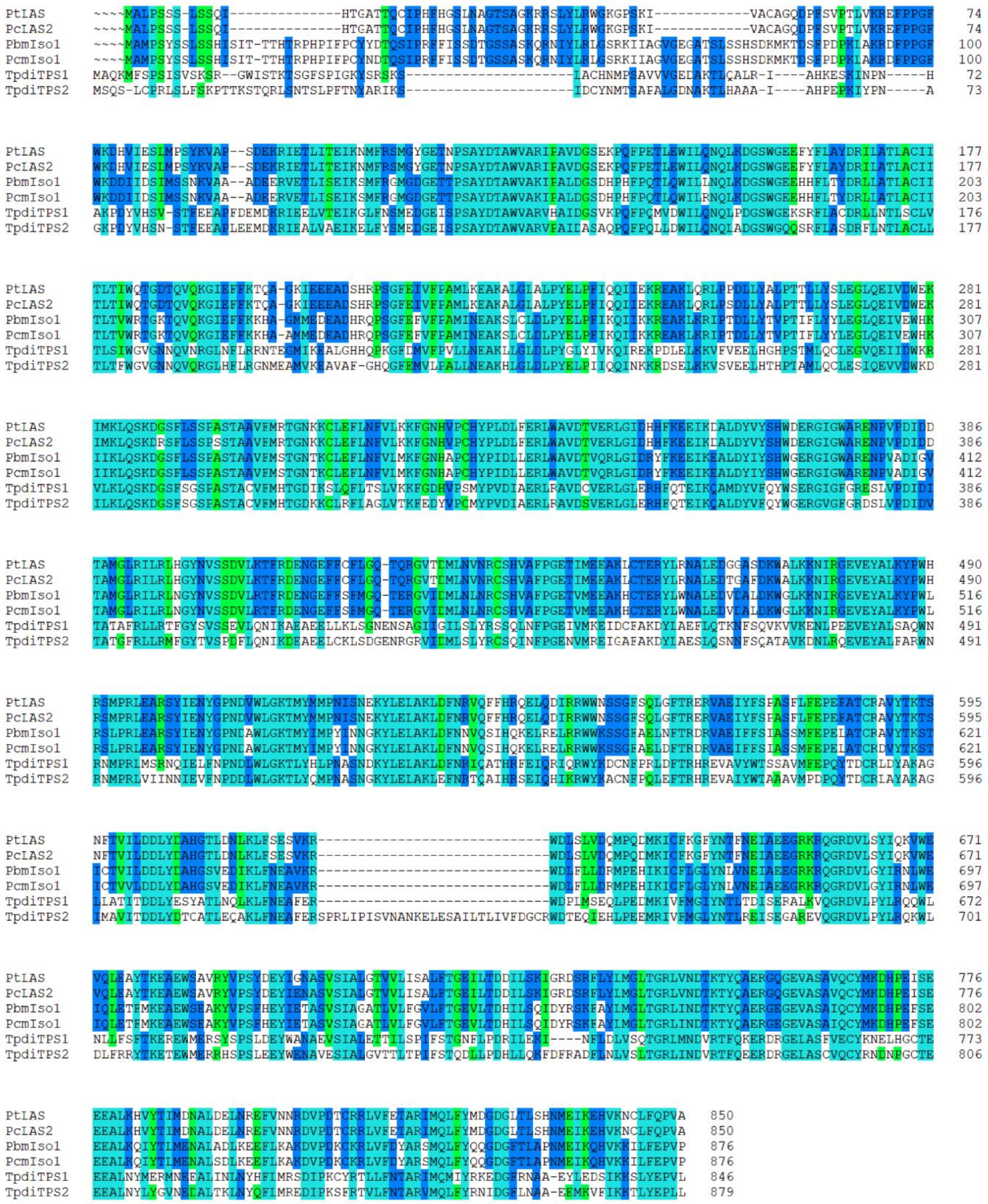

Supplemental figure S2. Amino acid alignment of *Thuja plicata* monofunctional diTPS1 (TpdiTPS1) and diTPS2 (TpdiTPS2) with *Pinus taeda* bifunctional levopimaradiene synthase (PtLAS, Q50EK2), *Pinus contorta* bifunctional levopimaradiene/abietadiene synthase (PcLAS2, JQ240311), *Pinus banksiana* (PbmIso1, JQ240313), and *Pinus contorta* (PcmIso1, JQ240314) monofunctional class I isopimaradiene synthase1.

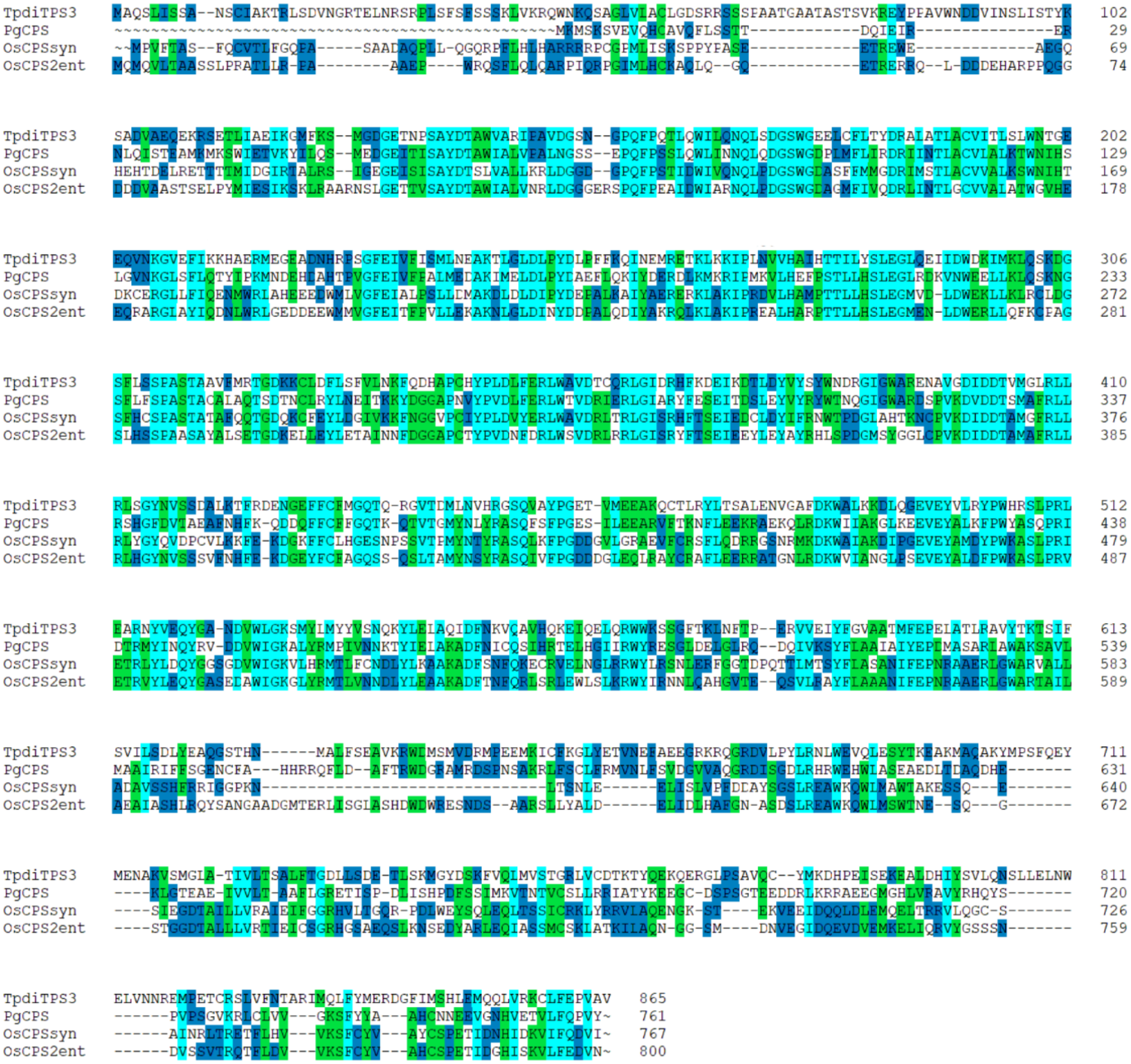

Supplemental figure S3. Amino acid alignment of *Thuja plicata* monofunctional diTPS3 (TpdiTPS3) with *Picea glauca* monofunctional *ent*-copalyl diphosphate synthase (PgCPS, GU045755), *Oryza sativa syn*-copalyl diphosphate synthase ($OsCPS_{syn}$, AY530101) and *Oryza sativa ent*-copalyl diphosphate synthase ($OsCPS2_{ent}$, AY602991).

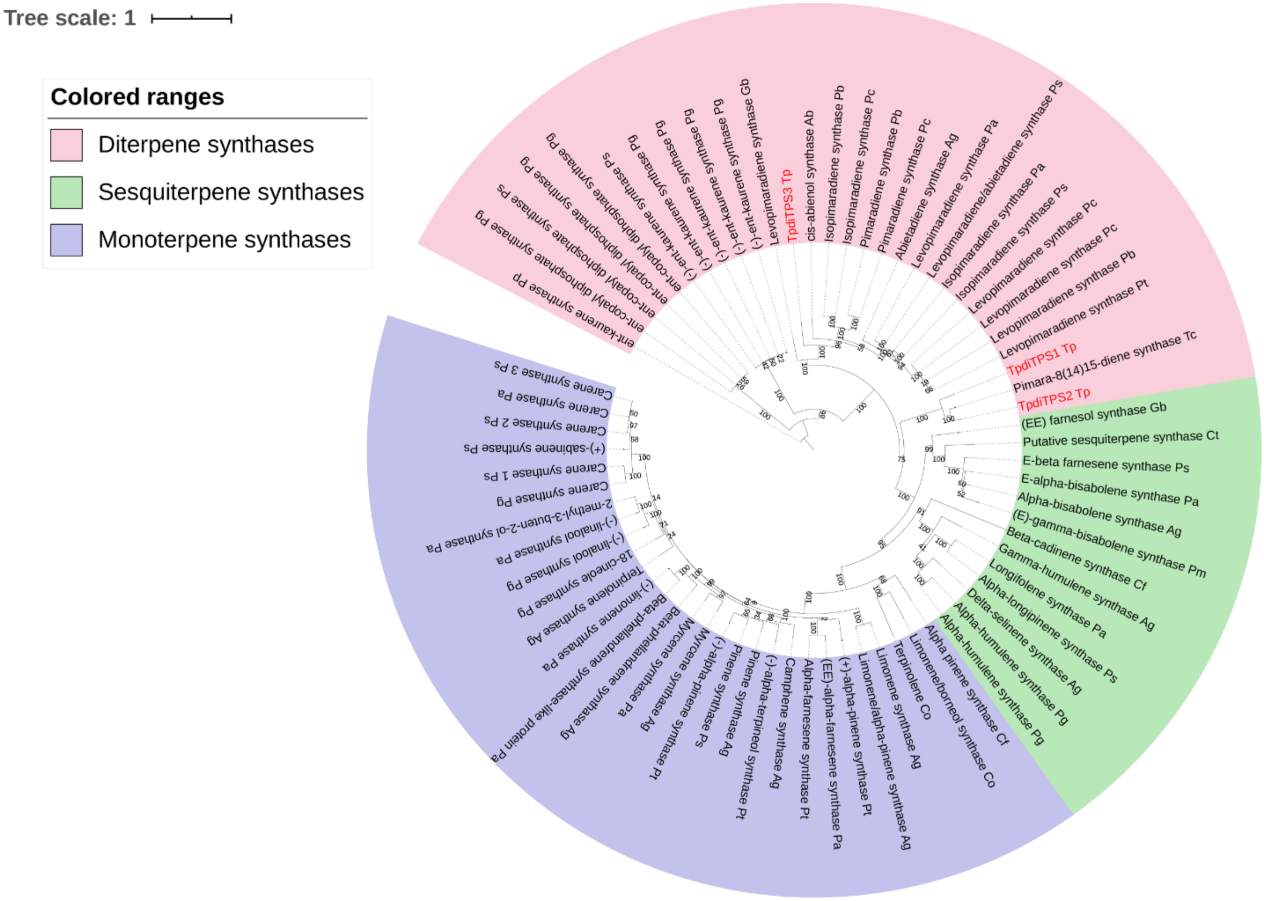

Supplemental figure S4: Phylogenetic relationships of conifer terpene synthases. The number of bootstrap replications was 100. The abbreviation of species are as follows: *Physcomitrella patens* (Pp) was used to root the tree. *Picea glauca* (Pg), *Picea sitchensis* (Ps), *Picea abies* (Pa), *Physcomitrella patens* (Pp), *Abies grandies* (Ag), *Pinus taeda* (Pt), *Pinus contorta* (Pc), *Pinus banksiana* (Pb), *Taiwania cryptomerioides* (Tc), *Abies balsamea* (Ab), *Chamaecyparis obtuse* (Co), *Chamaecyparis formosensis* (Cf), *Pseudotsuga menziesii* (Pm), *Cycas taitangensis* (Ct).

Supplemental Table S1: Primers used for nested PCR and SLIC-based cloning into expression plasmid.

Gene ID	*Primer set*	*Forward primer (5'-3')*	*Reverse primer (5'-3')*
TpdiTPS1	Set1	TCAAAATGGCTCAGAAGATGT	CCATGATTGTAATGATGCCTTTT
	Set2	TACTTCCAATCCAATGCAAAGACTCTTCAGGCTTTGAG	TTATCCACTTCCAATGTTATTACAGCACTGGTTCGTACA
TpdiTPS2	Set1	TACTCCCCATGGGATTCAAA	CACAGTTCATCCAATAGAGCAA
	Set2	TACTTCCAATCCAATGCAGCGAAAACACTTCACGCTG	TTATCCACTTCCAATGTTATTAGAGCAGGGGTTCATAGA
TpdiTPS3	Set1	CTGGTTTGGTTTTGGCTTGT	CCAAATCCCTCTCCAAGTGA
	Set2	TACTTCCAATCCAATGCAGCTGCCACTGCTTCTACATCG	TTATCCACTTCCAATGTTACTATACTGCCACTGGCTCGA

Supplemental Table S2: Kovats Retention indices of identified diterpene compounds

Gene Name	Compound Name	Retention index
TpdiTPS1	sandaracopimaradiene	1986
	syn-stemod-13(17)-ene	2119
	unknown diterpene	1981
TpdiTPS2	levopimaradiene	2039
TpdiTPS3	normal-copalol	2293
